# 465. Impact of Cancer on Clinical Outcomes of Dengue: A Matched Cohort Study in Colombia

**DOI:** 10.1093/ofid/ofaf695.156

**Published:** 2026-01-11

**Authors:** Silvio R Araujo, Valentina Galeano, Jorge Buitrago, Oscar Ramirez, Carlos A Portilla, Erika Cantor, Diana M Dávalos, Eduardo Lopez-Medina

**Affiliations:** Universidad del Valle, Cali, Valle del Cauca, Colombia; Centro de Estudios en Infectolgia Pediatrica CEIP, Cali, Valle del Cauca, Colombia; Clinica Imbanaco Grupo Quironsalud, Cali, Valle del Cauca, Colombia; Clinica Imbanaco Grupo Quironsalud, Cali, Valle del Cauca, Colombia; Clinica Imbanaco Grupo Quironsalud, Univerdidad del Valle, POHEMA Foundation, Universidad del Valle, Cali, Valle del Cauca, Colombia; Pontificia Universidad Javeriana, Centro de Estudios en Infectolgia Pediatrica CEIP, Cali, Valle del Cauca, Colombia; Centro de Estudios en Infectolgia Pediatrica CEIP, Cali, Valle del Cauca, Colombia; Centro de Estudios en Infectología Pediátrica CEIP, Departamento de Pediatría, Universidad del Valle, Clínica Imbanaco, Grupo Quironsalud, Colombia., Cali, Valle del Cauca, Colombia

## Abstract

**Background:**

With cancer prevalence rising, many patients in dengue-endemic regions face dengue exposure. Given the immune response’s role in dengue pathogenesis and limited data on outcomes in immunocompromised patients, this study assessed whether cancer affects the clinical features and outcomes of dengue.

**Figure:**
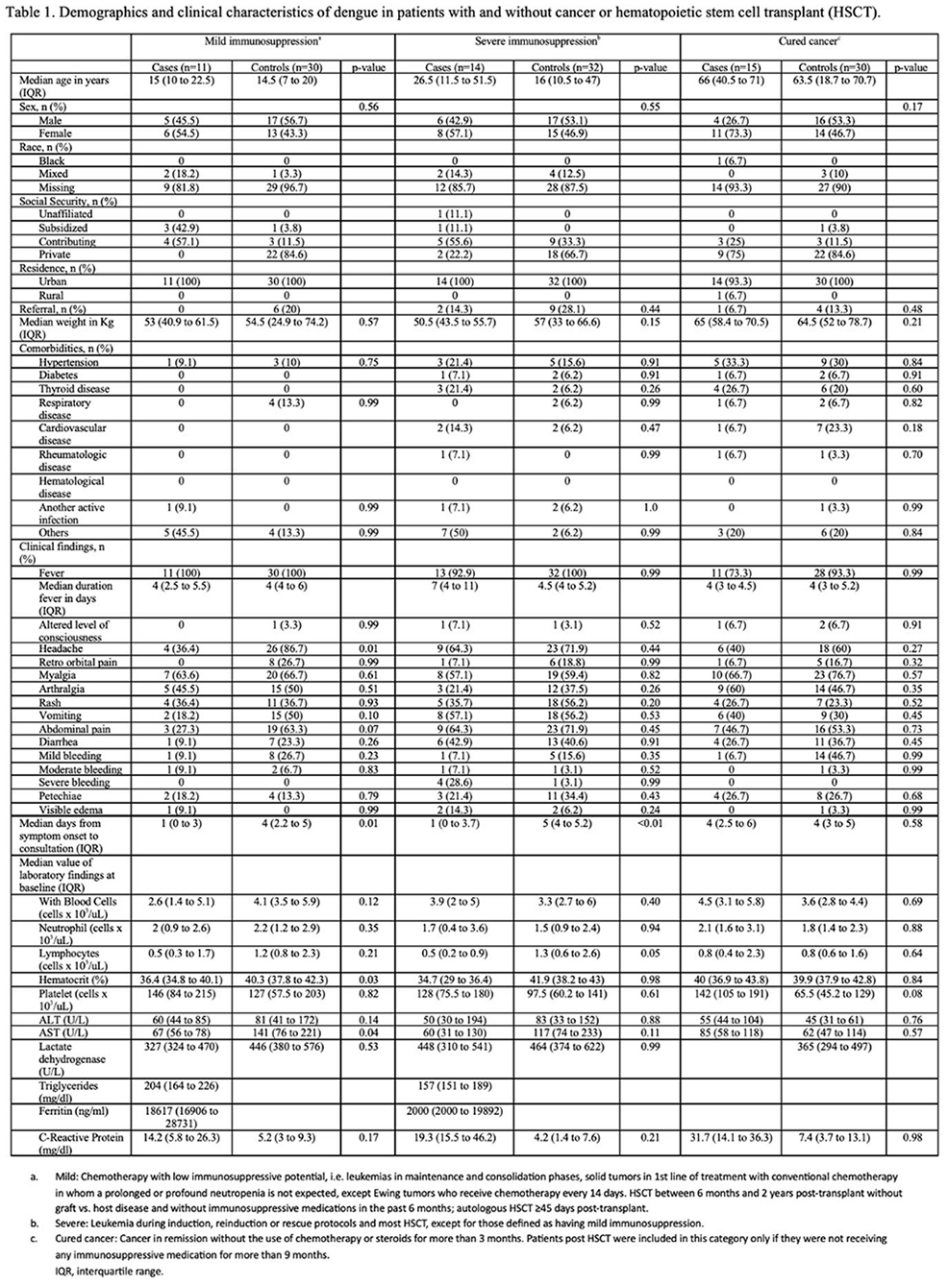

**Figure:**
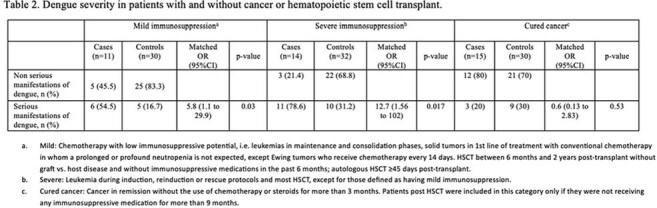

**Methods:**

Cohort of patients with virologically confirmed dengue (VCD) and cancer or hematopoietic stem cell transplant (cases), and age- and date-matched VCD controls without cancer, who consulted at Clínica Imbanaco, a referral hospital in Cali, Colombia, between Jan. 2016 and Dec. 2024. Patients were identified through the statistics department and their medical records reviewed. Cases were categorized as having mild or severe immunosuppression, or cured cancer, based on tumor type, transplant history, and date/type of chemotherapy or immunosuppression. The primary outcome was the occurrence of “serious manifestations of dengue”, defined as severe dengue (WHO 2009) or dengue with warning signs with a complicated course including vascular leakage (increases in hematocrit >20%, pleural effusion or ascites with respiratory compromise); bleeding with hemodynamic instability; thrombocytopenia < 20,000 or organ dysfunction. A matched univariate analysis using conditional logistic regression estimated odds ratios (ORs) for cases compared to controls.

**Figure:**
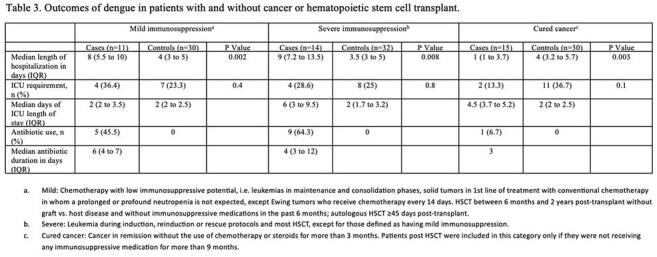

**Results:**

Forty cases (11 mild, 14 severe immunosuppression, 15 cured cancer) and 92 controls were identified. Demographic and clinical characteristics were similar, except cases sought consultation earlier (Table 1). Cases with mild and severe immunosuppression, but not those with cured cancer, had higher odds of “serious manifestations of dengue” (OR 5.77, 12.65, and 0.61, respectively) (Table 2). Cases with mild or severe immunosuppression had longer hospital stays and more frequent antibiotic use (Table 3).

**Conclusion:**

Immunocompetent patients and those with cured cancer similarly experience serious dengue manifestations. However, patients with cancer have an increased risk, which rises with greater immunosuppression. Medical personnel should educate cancer patients on dengue prevention while awaiting further data on vaccine safety and immunogenicity in this population.

**Disclosures:**

All Authors: No reported disclosures

